# How to Protect Fly Photoreceptors

**DOI:** 10.1371/journal.pbio.0040438

**Published:** 2006-11-28

**Authors:** Rachel Jones

When a nerve is injured, axons beyond the site of injury die through a process called Wallerian degeneration. This degeneration is delayed in mice that have a mutation called *Wallerian degeneration slow (Wlds)*; these mice have three copies of a particular stretch of their DNA. Because this piece of DNA includes the gene for nicotinamide mononucleotide adenylyltransferase (NMNAT), which synthesizes a molecule called NAD, there has been a great deal of interest in whether NMNAT or NAD can protect against axonal degeneration. Hugo Bellen and colleagues show that NMNAT can, at least in the fruitfly *Drosophila*—and that its protective ability is independent of its function as a NAD synthase.

Investigations into the putative protective role of NMNAT have produced conflicting results. In vitro evidence supports the idea that NMNAT protects against degeneration, as do studies in which NMNAT was overexpressed in *Drosophila*. But mice in which NMNAT is overexpressed show no protection.

The authors used a screening system that allowed them to look for flies that are homozygous for mutations—meaning that both copies of a gene are mutated—that result in death in cells of the visual system but are heterozygous, with one normal copy and one mutated copy, in the rest of the body. This means that mutations that would normally be lethal can be investigated in living flies. To look for mutations that affect synaptic function or development, the authors screened mutated *Drosophila* for abnormalities in phototaxis (a test of vision in which the organism moves toward or away from light; normal flies move toward light). They then tested the visual responses of the identified flies and looked for abnormal synapse structure in the eyes. This screening process identified two “nonsense” mutations in the gene for *Drosophila* NMNAT that prevents the gene from generating the correct protein.

**Figure:**
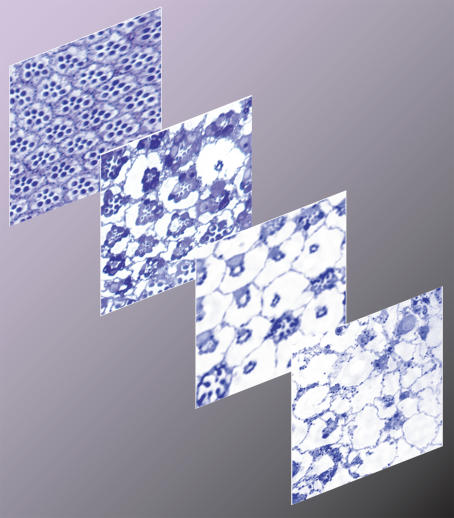
*Drosophila* photoreceptors that lack NMNAT degenerate very rapidly, as seen in this retina cross section that shows progressive degeneration from wild-type (in the back) to 28-day-old retina (in the front).

When they characterized the protein, the authors found that it was highly homologous to NMNAT proteins in humans and mice. Staining with an antibody against NMNAT showed that the protein is highly enriched in the fly nervous system, mainly in the cell nuclei and nerve terminals. Flies carrying the NMNAT mutation had abnormal photoreceptors that became progressively damaged with age, indicating a degenerative process. Mutant photoreceptors appeared to develop normally but did not survive. NMNAT therefore seems to be required for the maintenance of mature neurons.

What is the mechanism of degeneration in *nmnat* mutant photoreceptors? When mutant flies were raised in the dark, their degeneration was much less severe than when they were raised in the light, indicating that photoreceptor activity contributes to the degenerative process. Flies with double mutations that lacked both NMNAT and functioning photoreceptors also showed reduced neurodegeneration. This led the authors to conclude that the normal function of NMNAT is to protect photoreceptors against light-induced degeneration. They also showed that overexpression of NMNAT could protect photoreceptors against the degeneration caused by excessive activity in flies with mutations that cause continuous activation of photoreceptors.

To investigate the importance of NAD synthesis in neuroprotection by NMNAT, the authors generated an enzymatically inactive form of NMNAT. To their surprise, they found that expression of this protein could prevent neurodegeneration in *nmnat* mutants. However, this inactive NMNAT could not stop a full, homozygous *nmnat* mutation from being lethal to flies. These results indicate that NMNAT has two functions: its NAD synthase activity is essential for survival, but another activity is responsible for neuroprotection.

This study sheds new light on the mechanisms of neural degeneration and the functions of NMNAT. Attention will now turn to the identification of its second, non–NAD-dependent activity, as well as to continued attempts to reconcile the apparently conflicting results of earlier overexpression experiments.

